# Airway wall thickness is increased in COPD patients with bronchodilator responsiveness

**DOI:** 10.1186/s12931-014-0084-3

**Published:** 2014-08-08

**Authors:** Victor Kim, Parag Desai, John D Newell, Barry J Make, George R Washko, Edwin K Silverman, James D Crapo, Surya P Bhatt, Gerard J Criner

**Affiliations:** 1 Temple University School of Medicine, 785 Parkinson Pavilion, 3401 North Broad Street, Philadelphia 19140, PA, USA; 2 University of Iowa Hospital and Clinics, Iowa City, IA, USA; 3 National Jewish Health, Denver, CO, USA; 4 Brigham and Women’s Hospital, Boston, MA, USA; 5 University of Alabama, Birmingham, AB, USA

**Keywords:** Bronchodilator responsiveness, Airway wall thickness, Chronic obstructive pulmonary disease, Airflow obstruction

## Abstract

**Rationale:**

Bronchodilator responsiveness (BDR) is a common but variable phenomenon in COPD. The CT characteristics of airway dimensions that differentiate COPD subjects with BDR from those without BDR have not been well described. We aimed to assess airway dimensions in COPD subjects with and without BDR.

**Methods:**

We analyzed subjects with GOLD 1–4 disease in the COPDGene® study who had CT airway analysis. We divided patients into two groups: BDR + (post bronchodilator ΔFEV_1_ ≥ 10%) and BDR-(post bronchodilator ΔFEV_1_ < 10%). The mean wall area percent (WA%) of six segmental bronchi in each subject was quantified using VIDA. Using 3D SLICER, airway wall thickness was also expressed as the square root wall area of an airway of 10 mm (Pi10) and 15 mm (Pi15) diameter. %Emphysema and %gas trapping were also calculated.

**Results:**

2355 subjects in the BDR-group and 1306 in the BDR + group formed our analysis. The BDR + group had a greater Pi10, Pi15, and mean segmental WA% compared to the BDR-group. In multivariate logistic regression using gender, race, current smoking, history of asthma, %emphysema, %gas trapping, %predicted FEV_1_, and %predicted FVC, airway wall measures remained independent predictors of BDR. Using a threshold change in FEV_1_ ≥ 15% and FEV_1_ ≥ 12% and 200 mL to divide patients into groups, the results were similar.

**Conclusion:**

BDR in COPD is independently associated with CT evidence of airway pathology. This study provides us with greater evidence of changes in lung structure that correlate with physiologic manifestations of airflow obstruction in COPD.

## Introduction

COPD is classically associated with irreversible or poorly reversible airflow obstruction [1], which helps to distinguish it from asthma. However, the concept that asthma and COPD have a similar pathogenesis was initially described by Orie et al. in 1961 [2]. This concept, known as the “Dutch Hypothesis,” sparked tremendous debate, with many researchers arguing that COPD and asthma are distinct entities [3],[4]. Nevertheless, bronchodilator responsiveness (BDR) is present in 54-83% of COPD patients [5]–[7]. To complicate matters, this phenomenon in COPD is not a consistent one; BDR is present on some occasions but not others during serial lung function testing of the same subject [7],[8]. BDR may have significant clinical importance in COPD; some studies showed that BDR is associated with a faster rate of lung function decline [9],[10], mortality [11],[12], and the development of airflow obstruction [13], whereas others have not [14].

It is currently unclear why some patients with COPD demonstrate BDR. A crucial pathologic feature of COPD is airway inflammation, which results in epithelial remodeling, smooth muscle hypertrophy, subepithelial fibrosis, and mucous cell metaplasia [15]. Several investigators have correlated airway remodeling and degree of airflow obstruction in COPD [16]–[18]. Similarly, airway wall thickness on CT scan correlates with degree of airflow obstruction in several studies [19]–[21]. However, CT correlates of BDR in COPD have not been well described.

Given that BDR is characteristic of asthma and that asthma airway pathology is dominated by smooth muscle hypertrophy, it is reasonable to hypothesize that COPD patients with BDR have a greater degree of airway smooth muscle mass, and therefore thicker airways on CT imaging. We analyzed the 3661 subjects with COPD with quantified airway measurements in the Genetic Epidemiology of COPD Study (COPDGene®). We hypothesized that patients with BDR would have greater CT measures of airway pathology and less emphysema compared to those without BDR.

## Methods

The Genetic Epidemiology of COPD (COPDGene®) Study is a multicenter observational study to analyze genetic susceptibility for the development of COPD. This study was IRB approved at all institutions (Temple IRB #11369). Inclusion and exclusion criteria were described previously [22]. Briefly, enrollees are African-American or non-Hispanic Caucasian between the ages of 45 and 80 with at least a 10 pack-year smoking history. Exclusion criteria include pregnancy, history of other lung diseases except asthma, prior lobectomy or lung volume reduction, active cancer undergoing treatment, known or suspected lung cancer.

### Lung function testing and group assignment

We included patients with GOLD 1 through 4 disease severity in our analysis as defined by an FEV_1_/FVC ratio < 70. Each subject underwent pre- and post-bronchodilator spirometry using an EasyOne™ spirometer (Zurich, Switzerland) before and after the administration of 2 puffs of albuterol with spacer device. Bronchodilators were held prior to spirometry in usual fashion. Predicted values were obtained using NHANES III data [23]. BDR was assessed as an FEV_1_ % change after bronchodilator [(FEV_1_postBD – FEV_1_preBD)/FEV_1_preBD] × 100. 10% was used as a threshold of BDR. The patients were divided into two groups: BDR+, defined as ≥10% change in FEV_1_ [BDR + (10%)], and BDR-, defined as <10% change in FEV_1_ [BDR-(10%)]. Thresholds of ≥15% change and ≥12% and 200 mL in FEV_1_(American Thoracic Society criteria for BDR) were also analyzed separately as dividing points between groups [BDR + (15%) and BDR-(15%), BDR + (ATS) and BDR-(ATS)].

### Computed tomography

Volumetric CT acquisitions were obtained at full inspiration and at the end of normal expiration. Thin-slice collimation with slice thickness and intervals of < lmm was used to enhance spatial resolution. Quantitative image analysis to calculate lung volumes, percent emphysema, and percent gas-trapping was performed using 3D SLICER (http://www.slicer.org/). Percent emphysema was defined as the total percentage of both lungs with attenuation values < −950 Hounsfield units on inspiratory images, and percent gas trapping was defined as the total percentage of both lungs with attenuation values < −856 Hounsfield units on expiratory images. Total lung capacity and functional residual capacity were calculated based on inspiratory and expiratory CT images, respectively. Airway disease was quantified using VIDA Diagnostics Inc.’s PW workstation software (http://www.vidadiagnostics.com) as wall area percent (WA%: (wall area/total bronchial area)×100) [9]. The mean WA% was calculated as the average of six segmental bronchi in each subject. Using 3D SLICER, airway wall thickness was also expressed as the square root wall area of a 10 mm diameter airway (Pi10) and 15 mm diameter airway (Pi15) as previously described [21].

### Statistical analysis

Analysis was performed using SPSS v21.0. Categorical variables were compared between groups using Chi squared test. Continuous variables were evaluated using 2-tailed unpaired *t* test. Wilcoxon rank sum test was used for non-normally distributed data. A p value <0.05 was considered statistically significant. Pearson correlations were performed between the measures of airway wall thickness and percent change in FEV_1_. Multivariate logistic regressions were performed to assess the independent effects of airway dimensions on BDR, using the 10%, 15%, and ATS dividing points in separate models with current smoking, history of asthma, %emphysema, %gas trapping, FEV_1_ %predicted, and FVC %predicted as covariates. FEV_1_/FVC ratio was excluded due to significant co-linearity with spirometric parameters. In addition, a multivariate linear regression was performed for SGRQ scores, mMRC dyspnea scores, total and severe exacerbations as outcomes of interest with lung function and BDR as covariates.

## Results

There were 1306 subjects in the BDR + (10%) group (35.7% of total) and 2355 subjects in the BDR-(10%) group. Baseline subject characteristics are summarized in Table 1. The BDR + (10%) group was older, was less likely to be currently smoking, had a greater history of asthma, worse lung function, higher BODE indices, worse health related quality of life (SGRQ scores), greater dyspnea, and greater total and severe exacerbation frequency compared to the BDR-(10%) group. There were no differences in gender, race, BMI, or 6-min walk distance. Similarly, the BDR + (15%) (n = 798, 21.8% of total) group had a greater smoking history, history of asthma, higher BODE indices, worse SGRQ scores, greater dyspnea, worse lung function, and greater total exacerbation history compared to the BDR-(15%) group (n = 2863). However, unlike the differences between the BDR + (10%) and BDR-(10%) groups, the BDR + (15%) group had a lower 6-minute walk distance, and did not have a greater history of severe exacerbations (defined as an exacerbation requiring an emergency room visit or hospitalization) compared to the BDR-(15%) group. Similar to the BDR + (10%) and BDR + (15%) groups, the BDR + (ATS) group (n = 1276, 34.9% total) had worse lung function, higher SGRQ scores, BODE scores, and mMRC dyspnea scores, but there were no differences in total and severe exacerbations. In all groups, percent change in FVC was greater in the BDR + groups.

**Table 1:** Baseline characteristics

	**BDR-(10%)**	**BDR + (10%)**		**BDR-(15%)**	**BDR + (15%)**		**BDR-(ATS)**	**BDR + (ATS)**	
	**n = 2355**	**n = 1306**	**p**	**n = 2863**	**n = 798**	**p**	**n = 2385**	**n = 1276**	**p**
Age at enrollment	62.94 ± 8.43	63.55 ± 8.78	.039	63.04 ± 8.49	63.58 ± 8.83	.111	63.16 ± 8.36	63.15 ± 8.93	.963
Gender (% male)	57.4	55.1	.191	57.1	54.5	.185	56.8	56.1	.700
Race (% Caucasian)	79.1	79.9	.569	79.4	79.2	.905	79.6	78.9	.638
BMI (kg/m^2^)	27.71 ± 5.90	28.05 ± 6.03	.094	27.74 ± 5.93	28.16 ± 6.00	.073	27.71 ± 5.95	28.06 ± 5.95	.090
Smoking History (pack yrs)	51.72 ± 27.08	52.35 ± 27.18	.502	51.38 ± 26.74	53.98 ± 28.32	.017	52.05 ± 27.07	51.75 ± 27.03	.748
Current Smoking (%)	45.0	39.5	.001	43.8	40.4	.082	43.6	42.1	.401
Distance walked, ft	1262.57 ± 410.64	1237.53 ± 394.89	.077	1266.14 ± 409.31	1208.67 ± 387.07	<.0001	1253.44 ± 413.03	1254.04 ± 390.33	.966
History of Asthma (%)	21.3	27.6	<.0001	21.9	29.6	<.0001	22.2	26.2	.017
BODE index	2.25 ± 2.12	2.72 ± 2.05	<.0001	2.28 ± 2.10	2.94 ± 2.03	<.0001	2.35 ± 2.13	2.55 ± 2.05	.007
SGRQ score	34.49 ± 22.77	39.07 ± 22.47	<.0001	34.79 ± 22.87	40.91 ± 21.73	<.0001	35.22 ± 22.78	37.80 ± 22.65	.001
mMRC dyspnea score	1.80 ± 1.47	1.99 ± 1.43	<.0001	1.80 ± 1.47	2.11 ± 1.39	<.0001	1.82 ± 1.46	1.94 ± 1.45	.022
FEV1% pred	60.25 ± 23.51	53.90 ± 20.74	<.0001	59.82 ± 23.23	51.4 ± 19.63	<.0001	58.95 ± 23.24	56.17 ± 21.73	<.0001
FVC% pred	83.45 ± 20.52	80.88 ± 19.59	<.0001	83.37 ± 20.50	79.52 ± 18.91	<.0001	81.87 ± 20.35	83.77 ± 19.94	.007
FEV1/FVC	0.54 ± 0.13	0.50 ± 0.13	<.0001	0.53 ± 0.13	0.49 ± 0.13	<.0001	0.53 ± 0.13	0.50 ± 0.13	<.0001
FEV1% change	1.74 ± 6.46	20.10 ± 10.82	<.0001	3.60 ± 7.13	25.10 ± 11.22	<.0001	2.86 ± 7.50	18.43 ± 12.48	<.0001
FVC% change	2.94 ± 9.21	17.85 ± 20.58	<.0001	4.40 ± 9.85	22.13 ± 24.15	<.0001	1.43 ± 7.13	21.02 ± 19.78	<.0001
Exacerbation Frequency	0.59 ± 1.12	0.71 ± 1.22	.002	0.61 ± 1.14	0.70 ± 1.22	.047	0.61 ± 1.14	0.67 ± 1.19	.146
Severe Exacerbations	17.5	20.7	.018	18.1	20.4	.140	18.3	19.3	.476

Data presented as mean ± SD.

When a multivariate linear regression was performed for SGRQ scores, mMRC dyspnea scores, total and severe exacerbations as outcomes of interest with FEV_1_ %predicted, FVC %predicted, and BDR by either 10%, 15%, or ATS criteria as covariates, BDR was not a significant predictor for these outcomes. This indicates that the differences in dyspnea, health related quality of life, and exacerbations were more likely a consequence of the differences in lung function in the BDR + groups.

Quantitative CT measurements are summarized in Table 2. The BDR + (10%) group had slightly more %emphysema, had more %gas trapping, and a higher functional residual capacity compared to the BDR-(10%) group. Total lung capacity was not different between groups. Pi10, Pi15, and WA% were significantly greater in the BDR + (10%) group compared to the BDR-(10%) group. The results were similar with the BDR + (15%) and BDR-(15%) groups, but the differences was slightly greater compared to the differences between the BDR + (10%) and BDR-(10%) groups. The BDR + (ATS) group had greater Pi10, Pi15, and WA% compared to the BDR-(ATS) group. In contrast to the other two groups, the BDR + (ATS) group had similar %emphysema and a lower FRC compared to the BDR-(ATS) group.

**Table 2:** Quantitative CT measurements

	**BDR-(10%)**	**BDR + (10%)**		**BDR-(15%)**	**BDR + (15%)**		**BDR-(ATS)**	**BDR + (ATS)**	
	**n = 2355**	**n = 1306**	**p**	**n = 2863**	**n = 798**	**p**	**n = 2385**	**n = 1276**	**p**
%emphysema	11.36 ± 12.34	12.22 ± 11.96	.042	11.43 ± 12.23	12.53 ± 12.12	.024	11.78 ± 12.77	11.45 ± 11.96	.431
%gas trapping	33.96 ± 20.93	39.04 ± 20.22	<.0001	34.46 ± 20.85	40.48 ± 20.00	<.0001	34.93 ± 20.88	37.36 ± 20.61	.001
TLC	6.01 ± 1.43	6.09 ± 1.47	.090	6.01 ± 1.44	6.11 ± 1.48	.095	5.96 ± 1.44	6.31 ± 1.44	.134
FRC	3.78 ± 1.2	4.05 ± 1.24	<.0001	3.80 ± 1.20	4.15 ± 1.25	<.0001	3.87 ± 1.25	3.89 ± 1.10	<.0001
TLC %pred, race-adjusted	101.15 ± 16.52	103.65 ± 16.42	<.0001	101.34 ± 16.49	104.56 ± 16.44	<.0001	101.56 ± 16.41	102.94 ± 16.70	.016
FRC %pred, race-adjusted	117.90 ± 31.85	127.02 ± 31.12	<.0001	118.62 ± 31.69	130.25 ± 30.95	<.0001	118.86 ± 31.55	125.43 ± 32.10	<.0001
Pi10	3.69 ± 0.14	3.73 ± 0.15	<.0001	3.69 ± 0.14	3.74 ± 0.15	<.0001	3.69 ± 0.14	3.72 ± 0.15	<.0001
Pi15	5.17 ± 0.2	5.24 ± 0.21	<.0001	5.18 ± 0.20	5.26 ± 0.21	<.0001	5.17 ± 0.20	5.24 ± 0.21	<.0001
Wall area %, segmental	61.97 ± 3.15	63.12 ± 2.94	<.0001	62.08 ± 3.12	63.45 ± 2.89	<.0001	62.07 ± 3.14	62.97 ± 3.00	<.0001

Definition of *Abbreviations*: *TLC* total lung capacity, *FRC* functional residual capacity.

**Figure 1:**
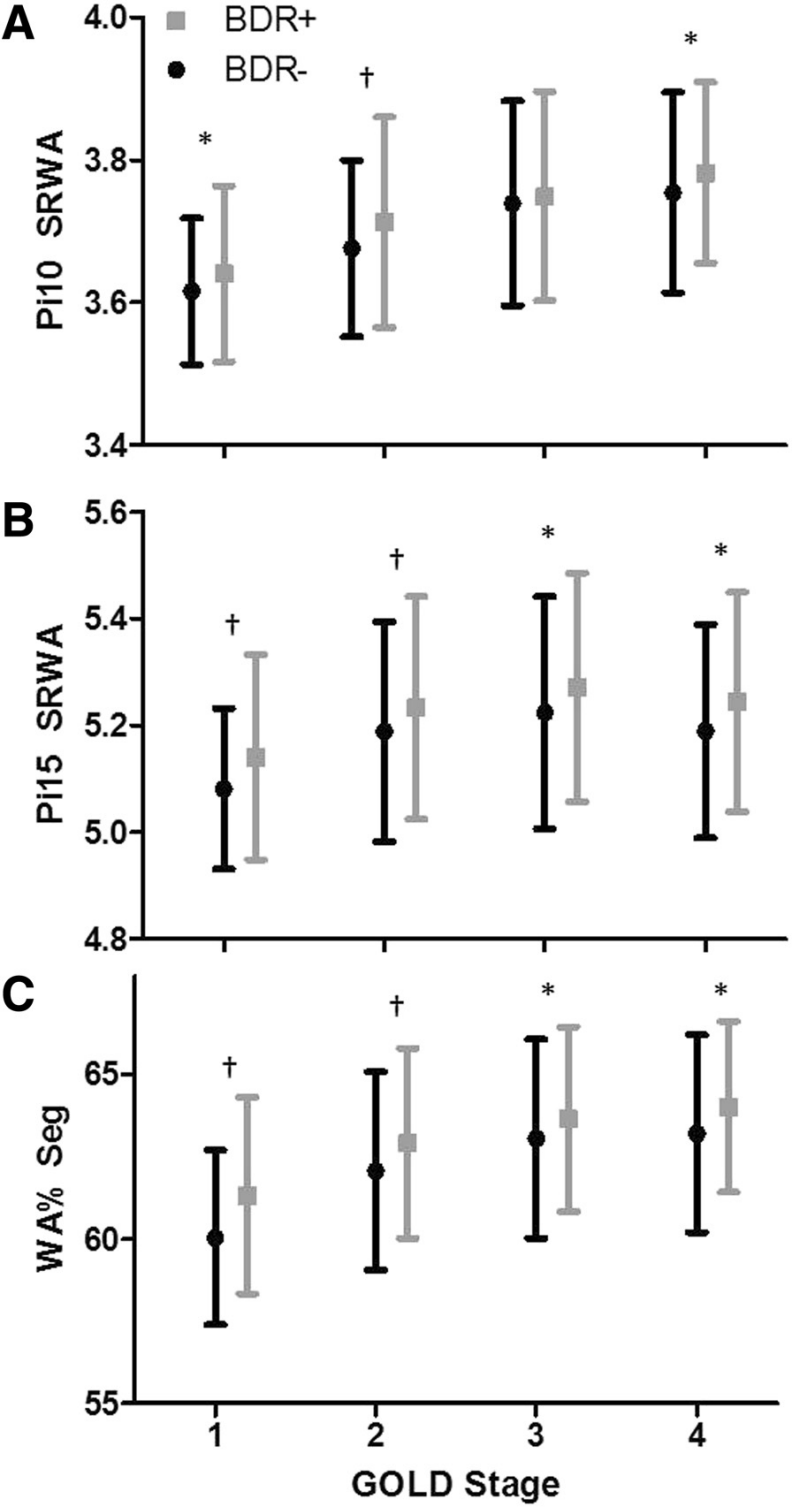
**Airway dimensions in those that are BDR + (10%) and BDR-(10%) according to GOLD stage.** Data presented as mean ± SD. **A)** Pi10, **B)** Pi15, and **C)** WA%. *p < 0.05. †p < 0.0001.

**Figure 2:**
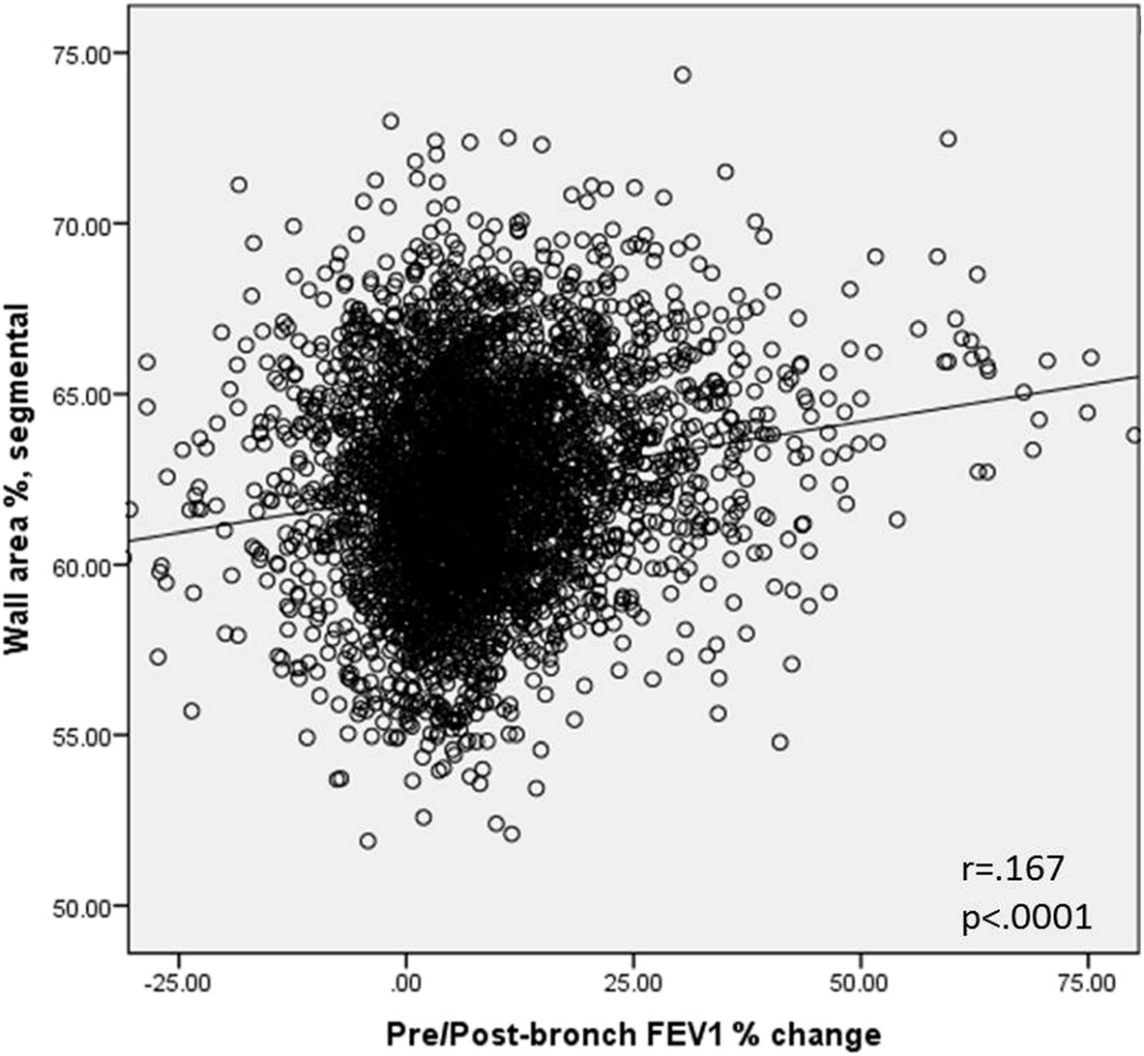
**Relationship between WA% and percent change in FEV**_**1**_**.**

Table 3 summarizes airway dimensions according to BDR + (10%) or BDR-(10%) by GOLD stage. In each GOLD stage, Pi10, Pi15, and WA% is greater in the BDR + (10%) group, with the exception of Pi10 in GOLD 3 subjects. This is depicted in Figure 1. The three variables for airway thickness were weakly related to percent change in FEV_1_ as a continuous variable. The correlation coefficients for Pi10, Pi15, and WA% are 0.148, 0.145, and 0.167, respectively, p < 0.0001 for all relationships. Figure 2 demonstrates the relationship between WA% and BDR.

**Table 3:** Airway dimensions by GOLD stage

**GOLD**	**Variable**	**BDR-(10%)**	**BDR + (10%)**	**p**
1		n = 519	n = 143	
	Pi10	3.62 ± 0.10	3.64 ± 0.12	.016
	Pi15	5.08 ± 0.15	5.14 ± 0.19	<.0001
	WA%, Seg	60.04 ± 2.66	61.32 ± 2.99	<.0001
2		n = 1029	n = 564	
	Pi10	3.68 ± 0.12	3.71 ± 0.15	<.0001
	Pi15	5.19 ± 0.21	5.23 ± 0.21	<.0001
	WA%, Seg	62.06 ± 3.01	62.90 ± 2.90	<.0001
3		n = 501	n = 425	
	Pi10	3.74 ± 0.14	3.75 ± 0.15	.289
	Pi15	5.22 ± 0.22	5.27 ± 0.21	.001
	WA%, Seg	63.04 ± 3.02	63.64 ± 2.81	.002
4		n = 506	n = 174	
	Pi10	3.75 ± 0.14	3.78 ± 0.13	.031
	Pi15	5.19 ± 0.20	5.24 ± 0.21	.004
	WA%, Seg	63.20 ± 3.01	64.02 ± 2.60	.003

Table 4 summarizes the odds ratios and 95% confidence intervals for the multivariate logistic regressions for the three measures of bronchodilator responsiveness (BDR ≥ 10%, BDR ≥ 15%, BDR + by ATS criteria) as the outcomes of interest with using the three measures of airway wall thickness as predictors of interest in separate models. All three measures of airway wall measures had significant odds ratios for bronchodilator responsiveness. The most significant odds ratios were for Pi15 (4.60 [95% CI 3.13, 6.75], 5.35 [95% CI 3.48, 8.24], and 5.13 [95% CI 3.46, 7.61] for BDR + (10%), (15%), and (ATS), respectively). Asthma history was not a significant predictor of BDR in these multivariate models (1.07 [95% CI 0.99, 1.15], 0.99 [95% CI 0.91, 1.08], and 0.97 [95% CI 0.81, 1.16] for BDR + (10%), (15%), and (ATS), respectively).

**Table 4:** Multivariate logistic regression for bronchodilator reversibility

	**BDR + (10%)**			**BDR + (15%)**			**BDR + (ATS)**		
	**OR**	**95% CI**		**OR**	**95% CI**		**OR**	**95% CI**	
**Pi10**	4.59	2.62	8.06	6.27	3.33	11.79	5.82	3.30	10.24
**Pi15**	4.60	3.13	6.75	5.35	3.48	8.24	5.13	3.46	7.61
**WA% Segmental**	1.11	1.08	1.14	1.12	1.09	1.16	1.10	1.07	1.13

Using each variable in lefthand column in a separate model with gender, race, current smoking, history of asthma, %emphysema, %gas trapping, FEV_1_ %pred, and FVC %pred as covariates. P < 0.001 for all odds ratios.

## Discussion

We have demonstrated that subjects with BDR have CT evidence of thicker airways than non BDR subjects using three different thresholds for change in FEV_1_. We also showed that the two groups divided by changes in FEV_1_ were similar in terms of race and gender distribution, but had higher BODE indices, worse lung function, worse health related quality of life and dyspnea, and greater exacerbation frequency. To our knowledge, this is the most comprehensive clinical and CT description of subjects with and without BDR.

We chose FEV_1_ to determine BDR using several cutoff values based on prior studies [24],[25] because of the notion that changes in FEV_1_ may be more reflective of airway disease. In addition to percent changes, we also analyzed an absolute change in FEV_1_ to divide subjects into groups. Indeed, many patients with COPD, particularly those with worse disease, demonstrate responsiveness to bronchodilator by an increase in FVC as opposed to FEV_1_[26]. This may represent decreases in air trapping and dynamic airway collapse during forced expiratory maneuvers [27]. In our study, we found that those with BDR by FEV_1_ criteria also had significantly greater increases in FVC, and thus could also be labeled “volume responders” in addition to “flow responders” [14].

Studies have shown conflicting results in clinical outcomes of BDR in COPD. There are some studies that show an accelerated decline in lung function in reversible patients, whereas other studies have not [9],[14],[28]. In the Evaluation of COPD Longitudinally to Identify Predictive Surrogate Endpoints (ECLIPSE), a three year prospective observational study, patients with BDR had a 17 ± 4 ml per year greater rate of decline in FEV_1_ compared to those without BDR [9], but no differences in mortality, hospitalizations, or exacerbations were found [29]. Bronchial hyper-responsiveness was found to accelerate lung function decline in over 7,000 individuals and conferred an odds ratio of 4.5 for the development of COPD [13]. However, Nishimura et al. categorized GOLD 1–4 COPD patients into rapid decliners, slow decliners, and sustainers of lung function and found no difference in the presence of BDR among these three groups [14]. Similarly, there was no relationship between BDR and rate of lung function decline in the Inhaled Steroids in Obstructive Lung Disease in Europe trial (ISOLDE) or the Understanding Potential Long-term Improvements in Function with Tiotropium trial (UPLIFT) [24],[28]. In addition, acute bronchodilator responsiveness did not predict improvements in lung function with tiotropium [24]. Therefore, it is unclear if BDR in COPD has any clinical significance.

Part of the problem lies in the variability in the definition of BDR across studies and the inconsistency of BDR in COPD subjects over time. Calverley et al. found that 52% of 660 patients with serial lung function testing switched responder status to bronchodilator [8]. In the National Intermittent Positive Pressure Breathing Trial, 65% of patients had at least 1 visit with 15% improvement in lung function with administration of isoproterenol over a period of 2 years [30]. Another complicating matter is that using American Thoracic Society criteria of a change in FEV_1_ of 12% and 200 cc may be too restrictive for subjects with very severe airflow obstruction [31]. For someone with an FEV_1_ of 800 cc, for example, a change of 200 cc would translate into a change of 25%, a change much greater than 12%. In addition, many patients with severe COPD demonstrate a change in FVC acutely, not necessarily FEV_1_, in response to bronchodilator [26]. Thus, a practical, consistent definition of BDR is needed in order to understand the importance of this physiologic phenomenon.

Regardless of its clinical consequences, BDR is associated with thicker airways based on quantitative CT imaging. The reason for these observed differences is not clear, but our results are hypothesis generating. It is possible that genetic influences have bearing on BDR in COPD, accounting for both the physiologic and CT differences between those with and without BDR. Indeed, one study revealed greater BDR in smokers of first degree relatives with early onset COPD [32], and another identified several genomic regions that could contain loci regulating BDR [33]. If that is the case, then this could mean there are different potential therapeutic targets for those with BDR. This would have to be confirmed with more extensive genetic analysis. In addition, we have shown that advanced emphysema patients with greater airway smooth muscle mass were more likely to demonstrate BDR on lung function testing [34]. Other investigators have found direct correlations with the degree of smooth muscle mass and airflow obstruction in COPD [16],[17]. The increased CT airway thickness in the BDR groups may represent a greater degree of smooth muscle hypertrophy, which is thought to be the pathologic correlate in asthma responsible for bronchodilator responsiveness and airway hyper-responsiveness. These findings, however, must be confirmed in studies that also obtain surgical lung tissue or autopsy obtained tissue to confirm our results pathologically. Finally, it has been proposed that gas trapping may be a surrogate marker for airway disease in COPD; the association between BDR and gas trapping in our cohort is consistent with this notion.

Of interest was the finding that percent emphysema and percent gas trapping were greater in those in the BDR + (10%) and BDR + (15%) groups, but there was no difference in emphysema in the BDR + (ATS) group. This most likely reflects the differences in lung function between the groups; FEV_1_ and FVC %predicted were lower in the BDR + (10%) and BDR + (15%) groups and higher in the BDR + (ATS) group. This phenomenon likely is a result of greater ease in achieving an absolute change of 200 mL with bronchodilator in those with better lung function. In addition, in the multivariate models for BDR + presented in Table 4, %emphysema had slightly but significantly reduced odds ratios for BDR, and %gas trapping had slightly but significantly greater odds ratios for BDR. This is consistent with prior studies that have found lower percent emphysema is associated with BDR [35].

This study has some noteworthy limitations. Firstly, lung function testing was only performed once, leading to the possibility of inconsistency and lack of repeatability of the response to bronchodilator during serial testing. Some subjects that were not reversible may demonstrate reversibility on their next measure of airflow by spirometry [8]. Secondly, outcomes such as exacerbation and asthma history were by subject self-report, lending to recall bias. In addition, the inclusion of subjects with asthma, whether or not the diagnosis was made by a physician, is a limitation of the study. Thirdly, the differences in airway measurements were small between groups, making the significance of the data questionable. However, the multivariate analyses clearly show BDR, using various definitions, to increase the odds of having thicker airways. Finally, many of the clinical phenotypic differences are most likely the result of differences in lung function between the two groups.

## Conclusions

We show that quantitative CT measures of airway thickness are greater in those with bronchodilator responsiveness. This is one more step towards bridging the gap between radiological and pathological correlations. How these differences in airway thickness translates to airway pathology remains to be determined.

## Abbreviations

BDR: Bronchodilator responsiveness

BDR + (10%): Bronchodilator responsiveness as defined by increase in FEV_1_ by 10%

BDR + (15%): Bronchodilator responsiveness as defined by increase in FEV_1_ by 15%

BDR + (ATS): Bronchodilator responsiveness as defined by increase in FEV_1_ by 12% and 200 mL

BODE: Body mass index, airflow obstruction, dyspnea, exercise capacity

COPD: Chronic obstructive pulmonary disease

CT: Computed tomography

FEV_1_: Forced expiratory volume in 1 second

FRC: Functional residual capacity

FVC: Forced vital capacity

mMRC: Modified Medical Research Council

Pi10: Airway of 10 mm internal perimeter

Pi15: Airway of 15 mm internal perimeter

SGRQ: St. George’s Respiratory Questionaire

TLC: Total lung capacity

WA%: Wall area percent

## Competing interests

The authors declare no competing interests.

## Authors’ contributions

VK, PD, GJC were involved in the conception, hypotheses delineation, and design of the study and acquisition of the data; VK, PD, GW, JDN, and GJC were involved in the analysis and interpretation of the data; VK, PD, GJC, SB, GW, BJM, JC, EKS were involved in the writing the article or substantial involvement in its revision prior to submission. VK is the guarantor of the content of the manuscript. All authors read and approved the final manuscript.
